# How early can we detect diabetic retinopathy? A narrative review of imaging tools for structural assessment of the retina

**DOI:** 10.1007/s00417-025-06828-3

**Published:** 2025-05-16

**Authors:** Megan Vaughan, Philip Denmead, Nicole Tay, Ranjan Rajendram, Michel Michaelides, Emily Patterson

**Affiliations:** 1 UCL Institute of Ophthalmology, University College London, London, UK; 2 Moorfields Eye Hospital NHS Foundation Trust, London, UK; 3 UCL Medical School, University College London, London, UK; 4 Occuity, Reading, London, UK

**Keywords:** Diabetes, Diabetic retinopathy, Detection, Imaging, Fundus photography, Optical coherence tomography

## Abstract

**Abstract:**

Despite current screening models, enhanced imaging modalities, and treatment regimens, diabetic retinopathy (DR) remains one of the leading causes of vision loss in working age adults. DR can result in irreversible structural and functional retinal damage, leading to visual impairment and reduced quality of life. Given potentially irreversible photoreceptor damage, diagnosis and treatment at the earliest stages will provide the best opportunity to avoid visual disturbances or retinopathy progression. We will review herein the current structural imaging methods used for DR assessment and their capability of detecting DR in the first stages of disease. Imaging tools, such as fundus photography, optical coherence tomography, fundus fluorescein angiography, optical coherence tomography angiography and adaptive optics-assisted imaging will be reviewed. Finally, we describe the future of DR screening programmes and the introduction of artificial intelligence as an innovative approach to detecting subtle changes in the diabetic retina.

**Clinical Trial Registration number:**

N/A

## Abstract (highlights)

**Key Messages:**

*****What is known***:**

- Diabetic Retinopathy is the leading cause of vision loss in working-age adults in the developed world.
- Standard imaging methods like fundus photography, optical coherence tomography (OCT), and fundus fluorescein angiography are essential tools in diabetic retinopathy assessment.

*****What is new***:**

- Newer imaging techniques, including adaptive optics-assisted imaging, offer higher resolution and better detection of subclinical changes in the diabetic retina.
- Artificial intelligence is emerging as a promising tool in diabetic retinopathy screening programs, potentially reducing healthcare costs and manual grading time, though further validation is needed for widespread integration.

## Introduction: An overview

Diabetes mellitus (DM) is *a chronic disease that occurs when the pancreas does not produce enough insulin, or when the body cannot effectively use the insulin it produces* [1], and is associated with many ocular complications, such as cataract [2], ocular surface disease [2] and retinal changes, known as diabetic retinopathy (DR) [3].

DR is a chronic and gradual complication of DM that can result in sight-threatening changes, and any patient diagnosed with DM is at risk of developing DR [4]. DR is the leading cause of vision loss in working-age adults in the developed world [5], and almost one million are classed as blind due to DR [6]. Due to the ageing and growing population, and as life expectancy of people with DM also rises, DR is likely to become more prevalent [7, 8], particularly as almost all people with Type I (T1) DM and at least 60% of people with Type II (T2) DM, will develop DR after 20 years from diagnosis [9]. Timely detection of DR is critical, especially as late detection contributes to poorer outcomes [10, 11]. There is an economic burden on healthcare systems due to the preventable complications associated with DM and DR [12] and, crucially, late detection is associated with higher costs than early detection [13]. Therefore, novel strategies to halt progression of DR could reduce this burden and allow the allocation of time and resources to other aspects of healthcare.

### Diabetic retinopathy

**Fig. 1:**
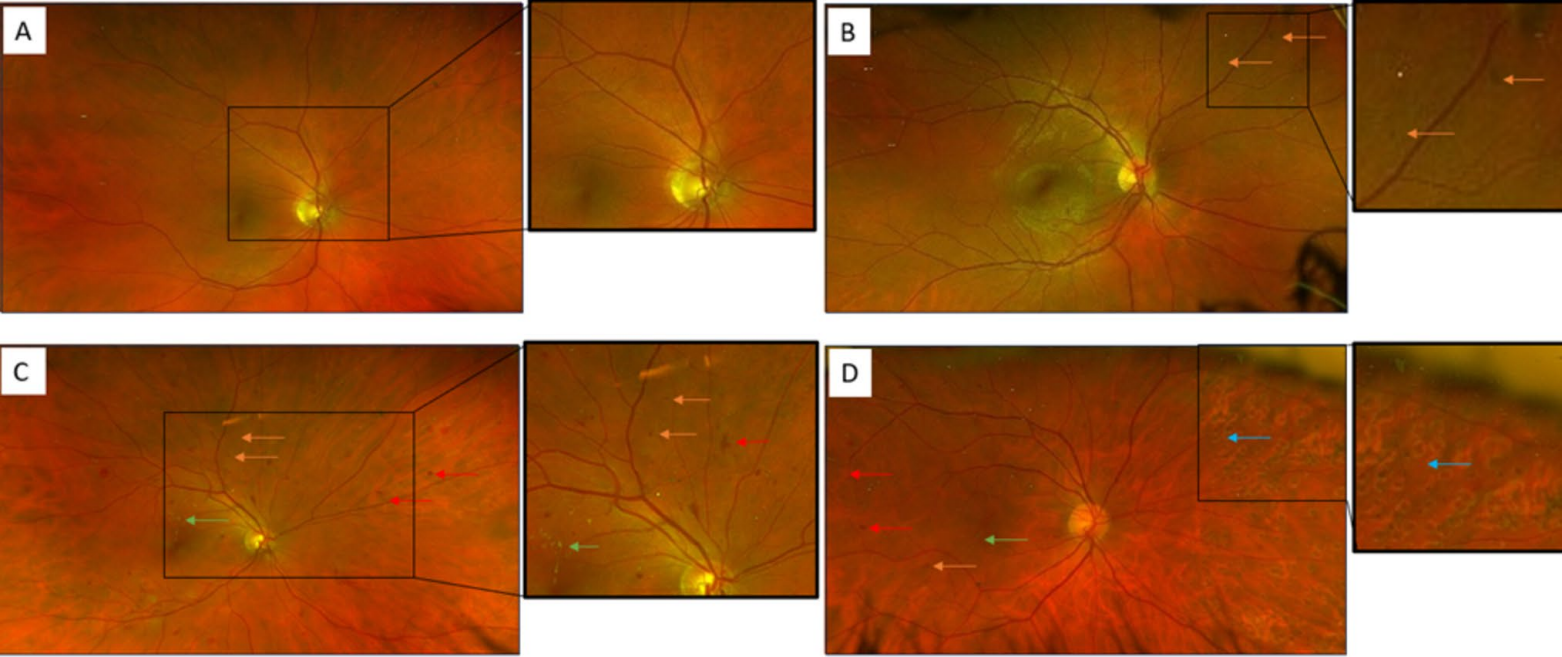
Fundus images from **A**) a normal control, **B**) mild non-proliferative diabetic retinopathy (DR) (R1), **C**) moderate non-proliferative DR (R2), and **D**) severe proliferative DR (R3) The arrows indicate dot haemorrhages (orange), blot haemorrhages (red), hard exudates (green) and scars from laser therapy for neovascular disease (blue)

Diabetic retinal disease can be characterised by two phenotypes: DR and diabetic macular oedema (DMO), with DR further subcategorised into: non-proliferative diabetic retinopathy (NPDR) and proliferative diabetic retinopathy (PDR), shown in Fig. 1. Characteristic signs of NPDR include microaneurysms (MA), dot and blot haemorrhages, exudates and cotton wools spots [14]. PDR is characterised by neovascularisation of the disc, the iris, and elsewhere, as well as fibrosis, leading to tractional retinal detachments. Typically, NPDR precedes PDR and both features are observed in PDR [15]. The risk of severe visual loss from PDR with “High Risk” characteristics (such as hyperglycaemia, hypertension, and dyslipidaemia) [8] is around 50% at five years if untreated, compared to around 5% if PDR is treated with panretinal photocoagulation laser[16].

### Diabetic maculopathy

DMO is a complication of DM that can lead to vision loss. DMO is the accumulation of exudative fluid at the macula and is a common cause of decreased vision [17]. It can occur in any stage of DR and progression is different for each individual, but the risk increases with DR severity [9, 18].

DR is traditionally seen as a microvascular disease [19]. However, recent insights suggest a shift from this view [12]. Hyperglycaemia and hyperlipidaemia may trigger inflammation and increase vascular endothelial growth factor (VEGF), resulting in damage to vascular endothelial cells, increased permeability of vessels, and angiogenesis [17]. DR occurrence is linked to disease duration, poor glycaemic control, and hypertension [20], with pregnancy also contributing [12]. However, such risk factors do not account fully for the onset or severity of DR or DMO. Some patients with well controlled glycaemic levels and hypertension still develop signs of DR, while others with poor control do not [21], indicating genetics might play a role [9, 22–24]. Despite this, the microvascular system remains crucial, as retinal microvascular circulation can help detect and monitor systemic complications, such as renal disease [25, 26] and cardiovascular events [27].

The commencement of systemic treatment prior to the progression of PDR or DMO is effective in slowing the progression of DR and reducing the risk of visual loss, as demonstrated in the Diabetes Control and Complications Trial (DCCT) [28].I It is important to intervene before complications of DR or DMO become irreversible [29].As, at present, the treatments available for DR are aimed at later stages of the disease, when vision may already be affected. Therefore, a more comprehensive understanding of the disease in its early stages is required to provide new and more effective preventative measures [12].

Key interventions for delaying or slowing the progression of DR and DMO comprise laser photocoagulation, anti-VEGF, and steroid intravitreal injections [30]. Although anti-VEGF treatment can be highly effective in some patients [31], others do not respond to the intra-ocular injections [32–34]. There are also risks associated with anti-VEGF treatment, such as retinal detachment, uveitis, or systemic side effects [35], highlighting the need to weigh up the relative risks associated with treatment and disease progression.

Key to effective management of DR, which may delay or even prevent advanced stages of the disease [36, 37], is early identification and timely intervention. Functional changes in vision can be detected clinically through psychophysical testing such as colour vision [38–40], visual acuity (VA) [41], contrast sensitivity (CS) [17], and electrophysiology [42, 43]. Although such psychophysical tests can provide valuable insight into the extent of functional deficit of DR [14], they tend to be non-disease-specific and subjective, precluding their use as standalone DR detection tests. Moreover, by the time patients become aware of functional deficits in vision, structural (potentially irreversible) damage may already have occurred [44].

Retinal imaging techniques aid in the early identification and monitoring of preclinical DR markers and provide insight into its pathogenesis. Many retinal imaging techniques are now used routinely in the clinic to screen and monitor diabetic eye disease, such as fundus photography, optical coherence tomography (OCT), OCT angiography (OCTA), and fundus fluorescein angiography (FFA). More recently, adaptive optics-assisted imaging has enabled single-cell visualisation of the retina, which may help to elucidate the complex signs associated with the pathophysiology of diabetic eye disease and ultimately detect DR in its earliest stages. This review will critically assess these state-of-the-art imaging techniques and their efficacy as tools for the early detection of DR, as well as consider the future of DR screening.

## Imaging of diabetic eye disease

### Fundus photography

Fundus photography is the main method employed by higher income countries for DR screening services [45–47] (Fig. 1). It is quick, non-invasive, and well tolerated by patients [48]. There are two main modalities when considering fundus photography: conventional fundus photography and colour scanning laser ophthalmoscopy (SLO). Conventional fundus photography uses a traditional camera with white light to capture colour images of the retina, providing a broad overview, but can be affected by media opacities, like cataracts. In contrast, colour SLO employs laser light of different wavelengths to scan the retina, producing high-resolution images with superior contrast and detail which are less impacted by opacities.

The quality of conventional fundus cameras has improved dramatically over the past two to three decades, with most now able to acquire images with resolution of approximately 20 megapixels [29]. As a result, colour fundus photography has shown greater sensitivity than both indirect and direct ophthalmoscopy techniques [20]. In addition, colour fundus photography and DR grading can be more cost effective than slit lamp ophthalmoscopy and alleviates the need for ophthalmology or optometrist consultation [49].

However, conventional fundus photography has limitations in its ability to assess structural DR changes. Firstly, it only allows for two-dimensional imaging of the fundus and therefore does not enable visualisation of the separate layers of the retina. As a result, oedema and neurodegenerative changes may be missed. Secondly, the earliest detectable DR-related signs using conventional fundus photography include MAs and, by the time of their appearance, damage has already occurred to the patient’s retina, which in most cases is irreversible [24]. Finally, colour fundus photography is limited by the system’s field of view (typically 45° single-field), which can neglect peripheral lesions associated with DR [50]. This is especially relevant to early detection of DR, as people with peripheral lesions – and more specifically, retinal ischaemia – are almost five times more likely to develop PDR [29, 51].

**Fig. 2:**
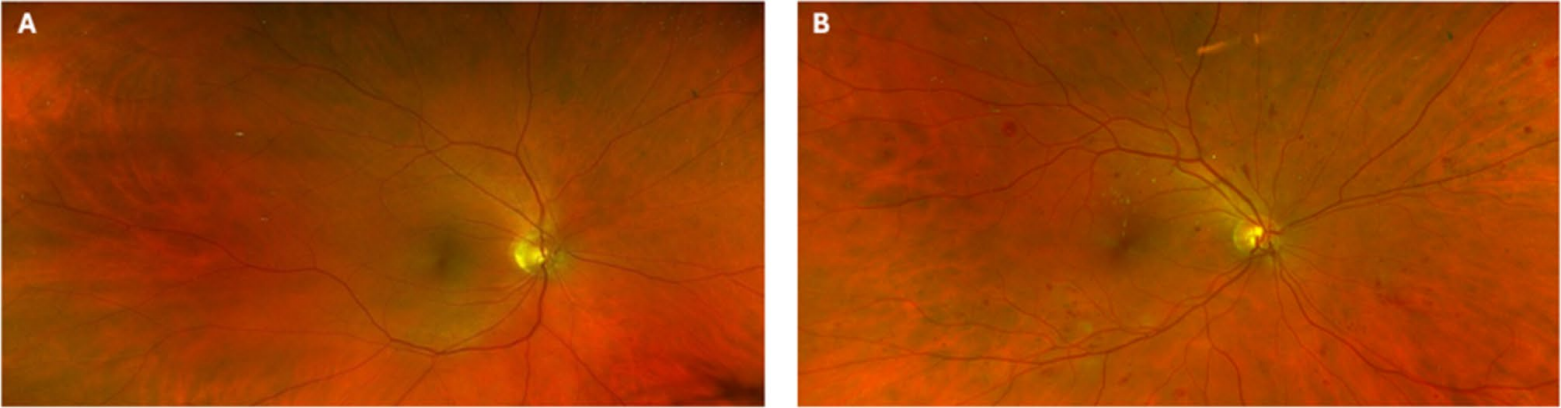
Ultrawide fundus image taken using an Optos system in **A**) a healthy retina and **B**) a retina with DR

Fortunately, colour SLO, widefield (angles greater than 50°) and ultra-widefield (UWF) (angles greater than 100°) imaging systems have become available in the past 20 years [51, 52]. Colour SLO and UWF systems offer superior colour imaging (Fig. 2) and allow clinicians to assess the peripheral fundus, even in undilated pupils, which has been suggested to be a predictor of DR progression over the following 4 years [53–55]. Furthermore, a study comparing conventional colour fundus photography with UWF found that DR was detected 17% more often in UWF [56]. Moreover, UWF helped to identify haemorrhages, MAs, venous beading, intraretinal microvascular abnormality, and neovascularisation in the periphery that would otherwise have been missed in the standard fundus imaging fields of view. Therefore, UWF may allow us to enhance the understanding of DR stages and progression, particularly as current definitions are based on standard colour fundus photographs, which may be outdated.

### Optical coherence tomography

**Fig. 3:**
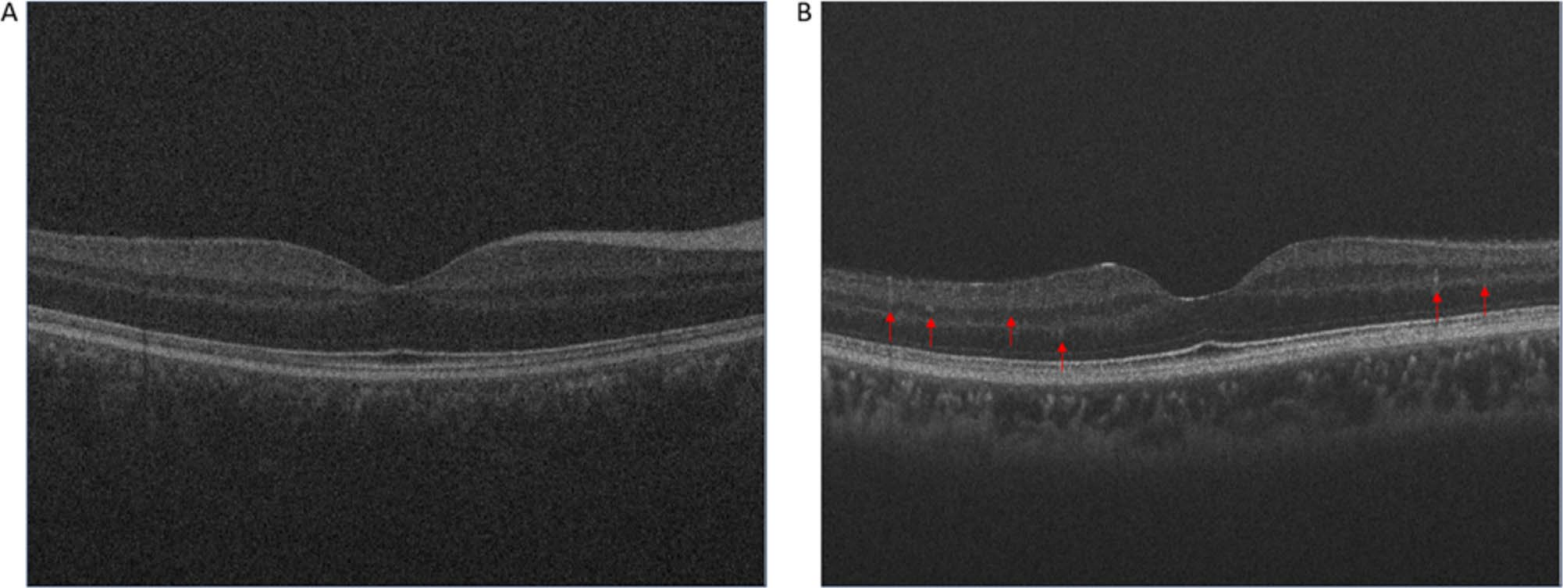
Optical coherence tomography (OCT) images **A**) healthy retina, and with **B**) moderate non-proliferative diabetic retinopathy (DR). The red arrows highlight hyperreflective intraretinal foci (HRF) common in patients with DR

OCT is another non-invasive technique that permits high-resolution imaging of the posterior pole’s anatomical structure. Like fundus imaging, OCT is quick and non-invasive. However, unlike fundus imaging, it provides a three-dimensional image of the retina (Fig. 3) [30, 51]. This enables transverse visualisation and assessment of both thickness [15] and morphology of the layers comprising the retina and choroid, which is of particular interest when investigating early changes in DR [48]. Advancements from time-domain OCT to the more sophisticated spectral-domain and swept-source devices has resulted in higher resolution and greater accuracy of thickness measurements [15]. Research has shown thinning of the RNFL and inner neural layers in people with DM, even when they do not have any other clinical signs of DR [37, 57, 58], making it a strong clinical structural assessment technique for the early detection of DR.

However, an important consideration when using OCT to measure retinal thickness is the differences between the variety of devices on the market. Research has shown that retinal thickness values vary according to the device used and are therefore not necessarily comparable when acquired using different systems. This makes it difficult to interpret results, especially in people with DR [59–61]. The variability can be due to inherent device differences in image acquisition, variable axial scaling, compression, motion artefacts, or the quality of the scan produced [62, 63]. These considerations are particularly pertinent when monitoring changes in a single patient in response to disease and/or treatment.

Finally, OCT features that are characteristic of early DR are not disease specific, which can make clinical decision making challenging. Retinal thinning or hyperreflective intraretinal foci (HRF) (as seen in Fig. 3B), for example, are associated with a number of other ocular conditions, such as glaucoma or age-related macular degeneration (AMD) [64, 65], so OCT is not yet appropriate as a sole method for early DR detection but should be incorporated into future DR screening programmes.

### Fundus fluorescein angiography

FFA is regarded as the gold standard for in-vivo and real-time evaluation of the structure of the retinal and choroidal vasculature [51, 57]. Clinically, FFA can be used as a guide for targeting specific retinal locations with laser treatment, and it enables detection of subtle changes in the retina, particularly vessel leakage, which is not seen using OCT. In particular, MAs, neovascularisation at the optic disc and elsewhere, peripheral areas of non-perfusion, and macular ischaemia may be more obvious using FFA than other imaging modalities [51, 66]. FFA is also particularly useful for differentiating intraretinal microvascular abnormalities from neovascularisation, which would be challenging to distinguish using fundus photography [51]. However, a major disadvantage of FFA is that it requires an intra-venous injection of sodium fluorescein dye and, with the advent of non-invasive OCTA, FFA may become a less favourable method of evaluating retinal health and the detection of DR.

### Optical coherence tomography angiography

**Fig. 4:**
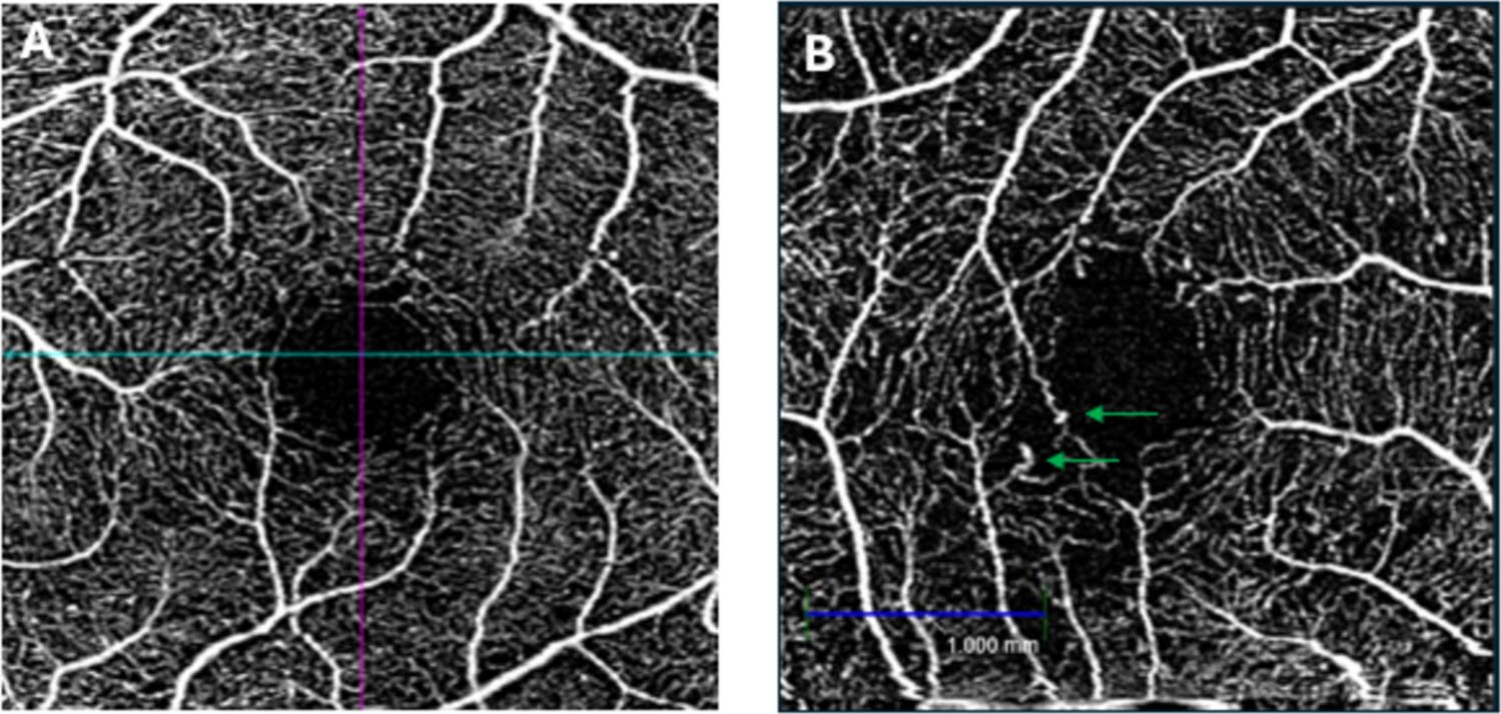
Optical coherence tomography angiography (OCTA) image of **A**) a healthy retina and **B**) with features of DR such as microaneurysms (green arrows)

In contrast to OCT, OCTA uses motion contrast [67], enabling non-invasive visualisation and objective quantification of both retinal and choroidal blood flow (Fig. 4) [36, 68, 69]. This gives it an advantage over FFA, as it eliminates the possibility of side effects from the intra-venous injection of sodium fluorescein dye, and can therefore be used in all patients, including in pregnancy [70]. Additionally, OCTA images are not disturbed by leakage of fluorescein or window defects [71]. OCTA has been shown to reveal several abnormalities that can be missed using FFA, such as areas of non-perfusion, greater vessel tortuosity and a decrease in the density of capillaries [72]. As a result, OCTA is considered to be particularly desirable over FFA as a clinical tool.

As with the imaging techniques already mentioned, OCTA can be used to identify MAs, intraretinal microvascular abnormality, neovascularisation, and sections of capillary non-perfusion [67, 73]. Additionally, it is possible to capture changes occurring in vessel density (VD), particularly in the capillary plexus (superficial, intermediate, and deep) [12, 51]. VD is the proportion of the vessel area with respect to the total area measured, and a decrease in VD occurs in both the superficial and deep capillary plexus in DR [51]. However, VD may not be the optimum metric for early diagnosis as, although VD is significantly reduced in patients with early signs of DR compared to healthy controls [73], no difference was found between controls and DM patients with no retinopathy, indicating that the decrease may only be detected once signs of DR are observed using other modalities.

However, when used to monitor changes in retinal capillary changes, OCTA has shown promise in its ability to detect people at risk of developing DR. Research has shown that retinal capillary changes occur before MAs are visible clinically [36], and OCTA can provide information on the expansion of the foveal avascular zone (FAZ) [73]. FAZ expansion is thought to result from capillary dropout, and it has been recognised that the FAZ diameter can enlarge with DR [36, 74]. In a meta-analysis carried out by Zhang et al. (2021), results clearly indicated an expanded FAZ area and decreased VD in the group with DM but no DR, compared to the healthy control group. However, when sub-categorised according to the type of DM (T1 or T2), microvascular alterations were negligible between T1 DM and the control group, while T2 DM remained significant. The authors postulate that this is due to people with T1 undergoing a ‘peaceful period’ after diagnosis, when OCTA would be unable to detect the preclinical signs of DR. Following this period, T1 patients then deteriorate promptly to NPDR, thus making it difficult for the device to detect these changes in a sufficiently timely manner [36]. Further research is required to determine if and why this occurs, especially since people with T1 DM have an increased prevalence of vision-threatening DR compared to people with T2 DM [75].

Widefield OCTA has useful applications, particularly when reviewing areas of retinal non-perfusion, as this finding tends to begin in the retinal periphery [76, 77]. Newer modalities using 12 × 12 mm field of view are valuable for vascular disorders of the retina, however, the larger field of view results in lower resolution of the microvasculature and so is not ideal for early detection of DR [71].

Additional shortcomings of OCTA are that it can be affected by artefacts and its small field of view. Projection artefacts occur when vessels that are more superficial appear in images of the deeper layers, and motion artefacts result from image displacement due to eye/head movements during acquisition [67]. Such artefacts are more common in OCTA than FFA, primarily due to the mode of image acquisition (i.e., scanning vs flood illumination respectively).

### Retinal oximetry

Retinal oximetry is a non-invasive imaging technique that allows assessment of retinal blood vessels’ oxygen saturation. The principle is similar to pulse oximetry (often using the finger or ear lobe) [78]; allowing for measurements of oxygen saturation across disease stage, but also across different regions of the retina [79]. It is known that retinal hypoxia can occur in DR and can often present early in the disease [80]. As retinal oximetry allows for visualisation of the blood supply and oxygenation of the blood, it may provide insights into the state of the retina prior to the observation of ischemia using other forms of imaging, like OCTA. Research has shown that oxygen saturation is altered in patients with DR [79]; there is, however, a lack of consensus (see [81] for a review), so it must be noted that it is still evolving as a technique. Although it shows strong repeatability, the results can be affected by the method of acquisition [82], and is therefore limited in its clinical use as a DR diagnosis tool at this time [83].

### Colour doppler imaging

Colour doppler imaging (CDI) utilises ultrasonography for non-invasive assessment and visualisation of the vessels in the retina, such as the ophthalmic artery and central retinal artery and vein [84, 85]. In the context of DR, CDI provides information about blood flow patterns, which have been shown to be impacted in people with DR [86]. Further, CDI has shown promise in its ability to identify patients at risk of developing DR [87, 88]. However, while CDI shows promise, its specific roles and clinical applications in DR detection and management are still being researched and refined [89].

### Fluorescence lifetime imaging ophthalmoscopy

Fluorescence lifetime imaging ophthalmoscopy (FLIO) is a relatively new non-invasive imaging technique used to study the retina in various retinal diseases, such as DR [90–92]. Many small molecules are naturally fluorescent and, when exposed to light, become excited to a higher electronic state. Upon returning to ground state, the molecule emits fluorescent light. FLIO measures both the fluorescence and the time spent in the excited state, providing detailed information about the metabolic and biochemical processes occurring in the eye, such as the accumulation of lipofuscin in DR [93]. Clinically, it can aid in the early detection of DR by identifying subtle metabolic abnormalities before visible structural changes manifest [92]. FLIO may also be able to contribute to monitoring disease progression and evaluating treatment responses, and investigation into the most promising molecular signatures for DR using this new technology is ongoing.

### Laser speckle contrast imaging

Laser speckle contrast imaging (LSCI) is another non-invasive imaging method that enables analysis of blood flow and perfusion. LCSI has previously been used to image organs, such as the liver and large intestine [94], and in diabetes for foot ulcers [95], but is relatively new to ophthalmology. Rodent models have demonstrated its use in the retina [96, 97], and its effectiveness in humans has also been shown [98, 99]. However, no study to date has used this to assess function in DR, and further research is warranted in this area.

### Adaptive optics-assisted imaging

**Fig. 5:**
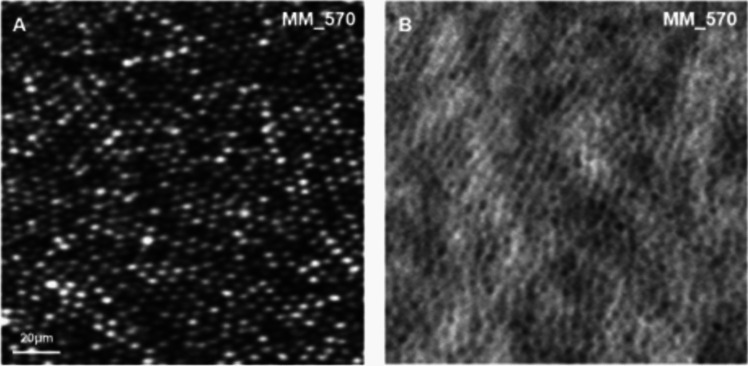
Confocal (**A**) and non-confocal split detection (**B**) AOSLO images of a healthy retina at ~1° temporal from the fovea

The detection of small lesions can be difficult to capture due to aberrations from the anterior part of the eye [100]. Higher order aberrations and astigmatism from an imperfect cornea or crystalline lens can result in wavefront aberrations, which makes the imaging of finer details in the retina near impossible with the imaging methods outlined above [30]. However, with the introduction of adaptive optics (AO)-assisted imaging, which can correct for such distortions, there are new opportunities to study different layers of the retina at a cellular level. AO technology enables acquisition of high-resolution retinal images in which it is possible to visualise individual cones and rods [101–104]. AO imaging devices correct for distortions using a wavefront sensor (Shack-Hartmann aberrometer), which calculates the ocular aberrations and then employs a wavefront correction (using a deformable mirror) to counteract these aberrations [48], (Fig. 5). Commercial AO devices, such as the rtx1™ AO retinal camera (Imagine Eyes, Orsay, France), can be used to visualise waveguiding cells, in addition to custom-built AO systems that can be modified according to the researchers’ needs.

### Adaptive optics scanning laser ophthalmoscopy

AO scanning laser ophthalmoscopy (AOSLO) is the most used form of AO-assisted imaging of the retina and comprises the majority of DR-related AO-assisted imaging data to date. AOSLO has variable depth of focus, enabling visualisation of abnormal features within different retinal layers, such as the nerve fibre layer and vasculature, making it a versatile tool to assess retinal structure in DR [105–108].

The most commonly used modality is confocal AOSLO (Fig. 5), which uses a pinhole to achieve diffraction-limited resolution. One drawback of confocal AOSLO is that only waveguiding or reflective structures can be visualised. Commercial systems utilise confocal imaging, whereas some custom-built systems have been modified to include other non-confocal imaging modalities. Non-confocal imaging modalities, such as split-detection, exploit light that is multiply scattered by the retina, which enables visualisation (for example) of the anterior end of cone inner segments, even in the absence of waveguiding cone outer segments [102, 109, 110]. The simultaneous acquisition of both modalities confocal and non-confocal images provides direct temporal correspondence, and coaxial alignment ensures direct spatial correspondence, between reflective structures (confocal) and underlying structure (split-detection).

Using AOSLO, researchers have demonstrated changes in retinal microvasculature in DR [111, 112], even in mild NPDR [113]. In addition, Karst et al. (2018) used both confocal and split-detection AOSLO to evaluate the appearance of the inner retinal layers within DR lesions and found changes in the thickness of vessel walls, as well as abnormal reflectivity and shadowing.

Research investigating the photoreceptor layer in DR is in its infancy but has shown great promise. In confocal images, healthy cones with intact outer segments are visible as bright spots, owing to their waveguiding properties. Cone density at the parafovea in T1 DM was found to be slightly reduced, using a commercially available (confocal) AOSLO system, and while cone density alone was not able to distinguish eyes with DR from eyes with no DR, a combination of cone metrics, proposed by Lombardo et al., did enable such differentiation [114, 115]. Regularity of the cone arrangement in both T1 and T2 DM has been associated with the presence of DR, increasing DR severity, and DMO using confocal AOSLO [116]. Recently, Elsner et al. (2022) demonstrated that cone waveguiding properties were altered in all ten DR patients assessed, but cone density was reduced in only five; despite all of the examined patients having total retinal thickness within normal limits for all quadrants, suggesting that AOSLO is a viable method for picking up early changes in the cone metrics of patients with DR.

AOSLO enables non-invasive visualisation of individual photoreceptors and tracking of individual cells across multiple time points to monitor natural history of disease [117]. However, using only confocal imaging, it is difficult to ascertain whether areas without bright spots are indicative of cone loss or simply altered waveguiding [118–120]. Additionally, there are changes in reflectivity that occur in the normal retina due to temporal fluctuation [121] and in response to light stimulation [122, 123]. It is therefore of interest to assess cone inner segment integrity using non-confocal imaging, although to-date no studies have done so.

### Adaptive optics optical coherence tomography

AO technology has also been applied to OCT [124], with AO-OCT imaging being used in a variety of retinal diseases, including DR [125, 126]. The primary benefit of AO-OCT imaging over other forms of imaging is that it can combine OCT’s ability to resolve depth and layers with high lateral resolution [127]. However, due to the limited availability of AO-OCT devices, and high failure rate compared to clinical OCT devices, AO-OCT in DR has not been explored fully. As in other coherent imaging devices (e.g., OCT), the AO-OCT is susceptible to speckle noise, which is further amplified by the higher magnification afforded by the AO element of AO-OCT [125, 128, 129].

AO-OCT has, however, been used to visualise areas of capillary of nonperfusion in eyes with NPDR and PDR [130]. These areas showed dramatic changes in cone morphology, particularly at the cone inner and outer segment junction and the cone outer segment tips, demonstrating the impact of capillary circulation on cone structure. However, only four people with DM (two with moderate DR and two with proliferative DR) were included in this study; larger, more representative data is therefore needed to draw meaningful conclusions about such changes in relation to DR stage.

Overall, AO-assisted imaging is showing great promise within the field of DR, with potential for use in the detection of DR at its earliest stages [100]. The usefulness of either AOSLO or AO-OCT will vary depending on the specific question being posed. In order to better understand the microvascular and photoreceptor anomalies detected with AO-assisted imaging devices, further research is required with larger cohorts of patients. To date, many studies have an uneven distribution of sex and type of DM and used small sample sizes [131]. In addition, cost, time consumption, reduced field of view, and the need for significant post-acquisition analysis all limit the application of AO-assisted imaging in a clinical capacity. It does, however, provide vital information about the retina in DM and subclinical DR.

## The future of screening for diabetic retinopathy

Subtle changes that occur in early DR are typically asymptomatic, so patients will often not present to the clinic until advanced complications (i.e., vitreous haemorrhage or tractional retinal detachment) develop. It is important that DR is detected early as, once these advanced complications occur, treatment outcomes are unfavourable [29]. Therefore, future research should focus on early detection and screening so that more can be learned about biomarkers that indicate which patients may benefit from early intervention (e.g., dietary advice) and to intervene before complications become irreversible. Taking baseline fundus images before onset of DR can serve as a reference point to track DR changes over time which can help future research understand the mechanisms of progression. Future work may focus on the emerging techniques outlined above to enhance the knowledge of early DR changes.

Regular screening assessments are a crucial aspect of successful diabetes care. DR screening models worldwide are focused on colour fundus photography examined by skilled graders [132, 133]. However, with the need for skilled graders to assess images [30], and with projections estimating that 700 million people will be affected with DM by 2045 [7, 8], DR screening services may be under significant strain to carry out manual grading of fundus photographs [134]. The incorporation of artificial intelligence (AI) in DR screening could facilitate timely treatment, reduce labour costs and save time spent on manual grading [30, 135]. AI software utilises algorithms, such as convolutional neural networks (CNN) in deep learning (DL), which uses pattern recognition to identify features related to DR. These algorithms are employed to interpret images through repeated analysis, which then compare the outcome to a benchmark (usually a manual grader) and can then correct itself if an error is made [29]. Many of these algorithms have high sensitivities and specificities, up to approximately 90% and 95% respectively, and a recent study has shown that they can perform with comparable or even superior accuracy than expert graders in multiple populations [136, 137]. Some modern DL systems have negative predictive values of roughly 99%, which translates to only a 1% likelihood of severe NPDR or PDR being missed [138].

A study that included more than 6,500 participants over a one-year period found that automated grading had similar effectiveness (based on sensitivity, specificity, and number of correct screening outcomes and cases referred appropriately) to manual methods and was less costly, posing a potential alternative to the current programme. In addition, there was a total of £201,600 in estimated savings to the NHS annually, with £4,088 in extra costs per additional case that was referable and £1,990 further costs per appropriate outcome (manual compared to automated) [139]. These findings highlight the usefulness of integrating AI automated systems into the current screening programmes, although there are various challenges regarding AI based algorithms when it comes to clinical application.

Many AI systems are still in their infancy and are under continuous development. In the case of ‘black box’ detection systems, using images previously graded for DR, little is known about the image information being used and how the output is determined. This is particularly limiting if the datasets used in the software are of a homogenous population, as there may be issues regarding generalisation [140]. Additionally, many algorithms are trained for use with a single disease. Previous studies have indicated that once confounding pathologies such as AMD, hypertensive retinopathy, artery or vein occlusions, and retinitis were introduced, the contemporary CNNs struggled with accurate diagnosis [141]. As such, the presence of co-pathology is likely to limit its utility in DR screening programmes.

With advancements in high resolution imaging techniques, such as OCTA and AO-assisted imaging, it may become possible to identify *patterns* of abnormalities that are disease-specific. With the ability to visualise microvasculature and individual photoreceptors, the limiting factor may no longer be the resolution, but rather the type of analysis performed. Quantification of retinal metrics often yields a single measurement (such as FAZ area, VD, cone density, etc.), which does not take full advantage of the rich information available within the image. For example, simply finding lower cone density in a retina will not enable a diagnosis of DR, but unique patterns of cone loss might. However, analysis of such granular detail, which is typically achieved manually, is time-consuming, so development of AI techniques for these high-resolution modalities is likely to yield significant benefits.

Finally, the ultimate goal of retinal imaging is not only to distinguish between those with DR and those without, but to accurately determine and monitor disease stage as well as risk. Non-invasive imaging techniques have a distinct advantage over more invasive methods (e.g. blood tests), as they are more tolerable for the patient, thereby facilitating regular screening and monitoring efforts. As such, it may be possible to detect not only DR (ocular disease) but also DM (systemic disease, i.e. oculomics). With almost one in two adults with DM being unaware of their condition [142], 9.1% having impaired glucose tolerance, and 5.8% having impaired fasting glucose worldwide [143], ocular screening for DM is a significant clinical target.

## Conclusion

This review has highlighted the advancements in imaging techniques in DR and their potential to improve clinicians’ ability to detect DR. A combination of functional and structural assessments is key to providing meaningful information about a patient’s DR status, and high-resolution structural assessments have a greater capacity to detect subclinical changes. Standard fundus photography is likely to remain the cornerstone of structural assessments, but with advancements in blood flow visualisation, OCT, and OCTA imaging, the use of these techniques is likely to grow. AO-assisted imaging has the potential to remodel the current approach in early detection and treatment of subclinical DR, due to its high resolution and capability of imaging different layers of the retina. Further, the introduction of AI to DR screening programmes shows promise, particularly with regard to the reduction of healthcare costs and time spent grading manually, but specificity may be limited if a patient has co-pathologies. Further validation will be needed before AI can be integrated into DR care at scale but, although it cannot replace expert grading, may provide a valuable tool for streamlining image analysis.
